# Double carbapenem as a rescue strategy for the treatment of severe carbapenemase-producing *Klebsiella pneumoniae* infections: a two-center, matched case–control study

**DOI:** 10.1186/s13054-017-1769-z

**Published:** 2017-07-05

**Authors:** Gennaro De Pascale, Gennaro Martucci, Luca Montini, Giovanna Panarello, Salvatore Lucio Cutuli, Daniele Di Carlo, Valentina Di Gravio, Roberta Di Stefano, Guido Capitanio, Maria Sole Vallecoccia, Piera Polidori, Teresa Spanu, Antonio Arcadipane, Massimo Antonelli

**Affiliations:** 1 Department of Anesthesiology and Intensive Care, Università Cattolica del Sacro Cuore, Fondazione Policlinico Agostino Gemelli, Rome, Italy; 2 Department of Anesthesia and Intensive Care, IRCCS-ISMETT (Istituto Mediterraneo per i Trapianti e Terapie ad alta specializzazione), Palermo, Italy; 3 Department of Laboratory Medicine and Advanced Biotechnologies, IRCCS-ISMETT (Istituto Mediterraneo per i Trapianti e Terapie ad alta specializzazione), Palermo, Italy; 4 Clinical Pharmacy, IRCCS-ISMETT (Istituto Mediterraneo per i Trapianti e Terapie ad alta specializzazione), Palermo, Italy; 5 Institute of Microbiology, Università Cattolica del Sacro Cuore, Fondazione Policlinico Agostino Gemelli, Rome, Italy; 6 Fondazione Policlinico A. Gemelli. Università Cattolica del Sacro Cuore, Largo A. Gemelli 8, 00168 Rome, Italy

**Keywords:** Double carbapenem, *Klebsiella pneumoniae*, Critically ill patients, Infections, Multidrug-resistant bacteria, Meropenem, Ertapenem

## Abstract

**Background:**

Recent reports have suggested the efficacy of a double carbapenem (DC) combination, including ertapenem, for the treatment of carbapenem-resistant *Klebsiella pneumoniae* (CR-*Kp*) infections. We aimed to evaluate the clinical impact of such a regimen in critically ill patients.

**Methods:**

This case–control (1:2), observational, two-center study involved critically ill adults with a microbiologically documented CR-*Kp* invasive infection treated with the DC regimen matched with those receiving a standard treatment (ST) (i.e., colistin, tigecycline, or gentamicin).

**Results:**

The primary end point was 28-day mortality. Secondary outcomes were clinical cure, microbiological eradication, duration of mechanical ventilation and of vasopressors, and 90-day mortality. Forty-eight patients treated with DC were matched with 96 controls. Occurrence of septic shock at infection and high procalcitonin levels were significantly more frequent in patients receiving DC treatment (*p* < 0.01). The 28-day mortality was significantly higher in patients receiving ST compared with the DC group (47.9% vs 29.2%, *p* = 0.04). Similarly, clinical cure and microbiological eradication were significantly higher when DC was used in patients infected with CR-*Kp* strains resistant to colistin (13/20 (65%) vs 10/32 (31.3%), *p* = 0.03 and 11/19 (57.9%) vs 7/27 (25.9%), *p* = 0.04, respectively). In the logistic regression and multivariate Cox-regression models, the DC regimen was associated with a reduction in 28-day mortality (OR 0.33, 95% CI 0.13–0.87 and OR 0.43, 95% CI 0.23–0.79, respectively).

**Conclusions:**

Improved 28-day mortality was associated with the DC regimen compared with ST for severe CR-*Kp* infections. A randomized trial is needed to confirm these observational results.

**Trial registration:**

ClinicalTrials.gov NCT03094494. Registered 28 March 2017.

**Electronic supplementary material:**

The online version of this article (doi:10.1186/s13054-017-1769-z) contains supplementary material, which is available to authorized users.

## Background

Over the last two decades, *Klebsiella pneumoniae*, one of the most common nosocomial pathogens, has accumulated many resistance mechanisms. After its first isolation in 1996, in the United States, carbapenem-resistant *Klebsiella pneumonaiae* (CR-*Kp*) strains have caused numerous outbreaks of nosocomial infections in the northeastern United States, Israel, Greece, and Italy, becoming a serious clinical issue in these areas [1–3].

CR-*Kp* isolates are spreading worldwide in an ongoing dynamic process, constituting a new global health threat. Infections related to this pathogen are associated with increased mortality, longer hospital and intensive care unit (ICU) stay, and consequent increased cost of care [4, 5].

Hospitalized patients with several comorbidities, poor functional status, and prior exposure to antibiotics are typically affected with such infections [6]. Preventive strategies aimed at reducing the spread of CR-*Kp* in the ICU are essential in reducing the infection rate related to these bacteria, which are a serious challenge for ICU physicians due to the exiguous available therapeutic options [7–10]. CR-*Kp* has an extensive drug resistance phenotype, and even the use of rescue antimicrobials (i.e., tigecycline, aminoglycosides, colistin, and fosfomycin) is frequently associated with clinical failure. Furthermore, although several reports suggest that the combination of more than one in-vitro active drug may be superior to monotherapy, the percentage of clinical failure remains high [11–14]. Conversely, among new antimicrobials, the use of novel beta-lactam/beta-lactamase inhibitor combinations (e.g., ceftazidime–avibactam) is still poor and is associated with the rapid emergence of resistance during treatment [15, 16].

Recently, the association of two synergistic carbapenems (ertapenem plus either meropenem, doripenem, or imipenem), alone or combined with other antibiotics, has been proposed as rescue treatment for CR-*Kp* infections [17–19].

Such combination therapy was prompted by evidence that ertapenem has the least in-vitro activity against CR-*Kp* due to an increased affinity for carbapenemases. Its role, as a suicide antibiotic, would allow the most active carbapenems to express their stronger activity at the infection site. However, although the data are encouraging, they are limited to in-vitro studies, case reports, and short case series [20, 21].

In this two-center, matched case–control study we analyzed the clinical and microbiological outcomes of patients affected with severe CR-*Kp* infections treated with a double carbapenem (DC) regimen, including ertapenem, compared with a standard rescue antibiotic treatment (ST).

## Methods

### Patient population and study design

This study was conducted in the general ICUs of two tertiary hospitals in Italy admitting approximately 1500 patients per year. We performed a retrospective 1:2 matched case–control analysis on data prospectively acquired from electronic ICU charts and computerized investigation of microbiology laboratory databases. Because of its observational, noninterventional design, the study was approved by the local ethics committees, which waived informed consent (approval numbers: UCSC14669/15; EC Palermo 2 n° 359/2015). Eligibility criteria were as follows: age ≥ 18 years; ICU admission between November 2012 and December 2015; documented CR-*Kp* invasive infection (i.e., pneumonia, bloodstream infection, complicated intra-abdominal, skin and soft tissue, and urinary tract infections); and targeted antibiotic therapy lasting ≥ 72 hours. Patients who received DC treatment, including ertapenem, were matched with those treated with other standard antibiotic combinations (ST). Matching was based on severity of illness as defined by the Simplified Acute Physiology Score (SAPS) II at ICU admission (within 5 points), and by the Sequential Organ Failure Assessment (SOFA) scores (within 2 points) upon diagnosis of infection [22, 23]. When multiple control candidates met the core matching criteria, the choice was based on ICU admission dates. Investigators were blinded to case outcomes during matching.

### Treatment regimens

Meropenem and ertapemen were administered every 8 hours and 12/24 hours, respectively, at a daily dose of 6 g and 2 g, respectively, adopting the extended infusion strategy (at least 3 hours) [24]. Colistin was administered every 12 hours at a daily dose of 9 million international units (MIU), after a 9 MIU loading dose. Gentamicin was administered every 24 hours at a daily per-kilogram dose of 5/7 mg. Tigecycline was administered at 100 mg every 12 hours after a 200 mg loading dose. Dosages were not changed during continuous renal replacement therapy (CRRT), including the colistin total daily dose, and were reduced in the presence of renal failure (except for tigecycline). Aminoglycoside therapeutic drug monitoring (TDM) was performed routinely.

### Definitions and outcomes

Ventilator-associated pneumonia (VAP), bloodstream infections (BSIs), intra-abdominal infections (IAIs), skin and soft tissue infections (SSTIs), urinary tract infections (UTIs), septic shock (SS) status, and acute respiratory distress syndrome (ARDS) were classified according to current guidelines [25–31]. The primary outcome end-point was 28-day mortality; secondary outcomes were clinical cure, microbiological eradication, duration of mechanical ventilation and of vasopressors, and 90-day mortality. Clinical cure was defined as the complete resolution of all signs and symptoms of the infection by the end of targeted therapy. Improvement or lack of progression of all abnormalities on chest radiographs was also required for VAP. Microbiological eradication was defined as the absence of the original pathogens from the culture of the specimens subsequently collected from the original site.

The empirical antimicrobial regimen (i.e., that used before in-vitro susceptibility data were available) was classified as inadequate when it did not include any agent displaying in-vitro activity against the isolated pathogen.

Clinical outcomes were independently evaluated by two physicians (GDP and GM) who were blinded to the type of treatment. When judgments were discordant (about 3% of patients), the reviewers reassessed the data and reached a consensus decision.

### Microbiological analysis

Strains were identified to the species level with matrix-assisted laser desorption ionization-time-of-flight (MALDITOF) mass spectrometry (MS) (BrukerDaltonik or Vitek MS; bioMérieux, Marcy l’Etoile, France). The antibiotic susceptibility profiling of isolates was carried out with the Vitek 2 system (bioMérieux). Results were interpreted according to the European Committee on Antimicrobial Susceptibility Testing (EUCAST) clinical breakpoints. Minimum inhibitory concentrations (MICs) of colistin and tigecycline were also assessed with the Sensititre broth microdilution method (Trek Diagnostic Systems, Cleveland, OH, USA). The phenotypic detection of carbapenemase expression in isolates with MICs > 1 mg/L for carbapenems was carried out according to EUCAST guidelines. Isolates screened as positive were further investigated by polymerase chain reaction to identify genes for class A carbapenemases (KPC and GES enzymes), class B metallo-β-lactamases (VIM, IMP, and NDM enzymes), and class D carbapenemases (OXA-23, OXA-24/40, OXA-48, OXA-51, OXA-55, OXA-58, and OXA-143) [32].

### Statistical analysis

The Kolmogorov–Smirnov test was used to evaluate the distribution of variables. Data with a nonnormal distribution were assessed with the Mann–Whitney test, and the median and selected centile (25th–75th) values are given. The data with a normal distribution were assessed with the Student’s *t* test. Categorical variables are given as proportions, and were analyzed with the chi-square test or Fisher’s exact test, as appropriate. *p* < 0.05 was considered significant. The crude odds ratio (OR) and 95% CI were calculated for each variable. We included all variables in the multivariable logistic regression if they reached *p* ≤ 0.1 on univariate analysis. A stepwise selection procedure was used to select variables for inclusion in the final model. The Hosmer–Lemeshow goodness-of-fit test and receiver operating characteristic (ROC) curve analysis were used to assess the goodness of the logistic final model. The Kaplan–Meier method was used for the survival analysis. All statistical analyses were performed using SAS 9.4 software (SAS Institute Inc, Cary, NC, USA).

## Results

### Population characteristics and treatment

During the study period, 243 patients with CR-*Kp* infection were identified. Among 72 potentially eligible case patients receiving a DC regimen including ertapenem, alone or in combination with another “in vitro active antibiotic” (mainly colistin), 24 patients were excluded (mainly because they were treated for less than 72 hours) and 48 patients were selected for the analysis (Additional file 1: Figure S1). These patients were compared with 96 corresponding controls who received an ST regimen not including carbapenems, matched for SAPS II and SOFA scores (baseline characteristics presented in Table 1).

**Table 1:** Demographic and clinical characteristics of the 144 patients with KPC *Kp* invasive infection included in the study

Variable	Number (%) of patients		*p* value
	DC group (*n* = 48)	ST group (*n* = 96)	
Demographics and comorbidities			
Age (years), mean ± SD	55.5 ± 15	61.3 ± 12	0.06
Males	35 (72.9)	58 (60.4)	0.19
CHF	18 (37.5)	27 (28.1)	0.34
COPD	11 (22.9)	12 (12.5)	0.17
CRF	8 (16.7)	7 (7.3)	0.15
Diabetes	17 (35.4)	31 (32.3)	0.85
CLD	10 (20.8)	8 (8.3)	0.06
Neoplasm	2 (4.2)	12 (12.5)	0.21
Immunosuppressive status	19 (39.6)	26 (27.1)	0.18
ICU stay before infection (days), median (IQR)	11 (4–24)	7 (1-22.5)	0.08
Duration of MV before infection (days), median (IQR)	6 (1.5-15.5)	5 (1–14)	0.57
Duration of vasopressors before infection (days), median (IQR)	3 (1–10)	1.5 (0–6)	0.11
Presenting feature			
Medical admission	25 (52.1)	59 (61.5)	0.37
Surgical admission	18 (37.5)	30 (31.3)	0.28
Trauma admission	5 (10.4)	11 (11.5)	0.92
SAPS II score, median (IQR)	44 (36–56)	46 (36–57)	0.56
SOFA score at infection, median (IQR)	9 (7–11)	8 (6–10)	0.33
Septic shock on occurrence of infection	36 (75)	43 (44.8)	**<0.01**
ARDS on occurrence of infection	18 (37.5)	23 (23.9)	0.13
CRRT on occurrence of infection	20 (41.7)	26 (27.1)	0.13
PCT on occurrence of infection (ng/ml), median (interval)	6.1 (3.2–50.4)	3.1 (0.8–5.9)	**<0.01**
Type of infection			
Pneumonia	25 (52.1)	49 (51)	0.95
IAI	9 (18.7)	10 (10.4)	0.19
SSTI	1 (2.1)	11 (11.5)	0.06
UTI	3 (6.2)	9 (9.3)	0.75
CVC BSI	8 (16.7)	10 (10.4)	0.42
Primary BSI	6 (12.5)	10 (10.4)	0.92
Secondary BSI	23 (47.9)	26 (27.1)	**0.02**
Multiple site infection	4 (8.2)	3 (3.1)	0.54
Therapeutic aspects			
IIAT	13 (27.1)	40 (41.7)	0.16
Overall duration of treatment (days), median (interval)	17 (11.5–25.5)	11.5 (7.5–15.5)	**<0.01**
Clinical and microbiological outcome			
28-day mortality	14 (29.2)	46 (47.9)	**0.04**
90-day mortality	24 (50)	58 (60.4)	0.31
Clinical cure	30 (62.5)	47 (48.9)	0.17
Microbiological eradication^a^	22 (50)	31 (38.3)	0.27
Duration of MV after infection (days), median (interval)	14 (7.5–31)	11.5 (7–19)	0.16
Duration of vasopressors after infection (days), median (interval)	10.5 (3.5–27.5)	8 (4.5–12)	0.19

Data presented as *N* (%), unless otherwise indicated. Bold data are significant

*IQR* interquartile range, *KPC Klebsiella pneumoniae* carbapenemase, Kp *Klebsiella pneumoniae*, *DC* double carbapenem, *ST* standard treatment, *SAPS* Simplified Acute Physiology Score, *SOFA* Sequential Organ Failure Assessment, *ICU* intensive care unit, *MV* mechanical ventilation, *ARDS* acute respiratory distress syndrome, *CRRT* continuous renal replacement therapy, *PCT* procalcitonin, *CHF* chronic heart failure, *COPD* chronic obstructive pulmonary disease, *CRF* chronic renal failure, *CLD* chronic liver disease, *IAI* intra-abdominal infection, *SSTI* skin and soft tissue infection, *UTI* urinary tract infection, *CVC* central vascular catheter, *BSI* bloodstream infection, *IIAT* initial inappropriate antimicrobial therapy

^a^Microbiological outcome was analyzed in 125 patients: 44 patients (DC group) and 81 patients (ST group)

There were no significant between-cohort differences in terms of demographics, type of admission, presenting features, main comorbidities, and infection types. Duration of ICU stay and the use of mechanical ventilation and vasopressors before infection occurrence were similar in both groups. About 50% of the patients were affected with CR-*Kp* pneumonia: 51 patients were classified as VAP, and 23 patients as hospital-acquired pneumonia (HAP) requiring ICU admission due to respiratory failure. Septic shock upon occurrence of infection, along with secondary bacteremia and high procalcitonin levels, were significantly more frequent in patients receiving DC treatment. The overall duration of therapy was significantly longer in the DC group than in controls (17 days vs 11.5 days, *p* < 0.01). Seven patients (4.9%) had multiple site infections, and the overall rate of initial inappropriate antibiotic therapy was less than 50% in both groups. Adjunctive nebulized colistin (2 MUI every 8 hours, via jet/ultrasonic nebulizers) was used in 32 patients with VAP (seven cases and 25 controls). No between-group differences were identified in terms of MV and vasopressor duration after infection. No adverse events potentially associated with the use of the DC regimen were observed in both centers.

In 23 patients (16%) the infection was polymicrobial (15 CR *Acinetobacter baumannii* isolates and eight CR *Pseudomonas aeruginosa* isolates), without statistically significant differences between the two groups. The rate of combination therapy including at least one other active antibiotic was higher in the DC group (72.9% vs 54.2%, *p* = 0.05): antibiotic association details for the DC and ST groups are presented in Table 2.

**Table 2:** Therapeutic and microbiological details according to the type of treatment

Variable	Number (%) of patients		*p* value
	DC group (*n* = 48)	ST group (*n* = 96)	
Polymicrobial infection	6 (12.5)	17 (17.7)	0.57
CR *Acinetobacter baumannii*	3 (6.25)	12 (12.5)	0.4
CR *Pseudomonas aeruginosa*	3 (6.25)	5 (5.2)	0.89
Combined targeted therapy	35 (72.9)	52 (54.2)	**0.05**
DC + colistin	19 (39.6)	–	**–**
DC + gentamicin	8 (16.9)	–	**–**
DC + tigecycline	3 (6.25)	–	**–**
DC + colistin + tigecycline	2 (4.2)	–	**–**
DC + colistin + gentamicin	3 (6.25)	–	**–**
Colistin + tigecycline	–	22 (22.9)	**–**
Colistin + gentamicin	–	13 (13.5)	**–**
Gentamicin + tigecycline	–	10 (10.4)	**–**
Colistin + tigecycline + gentamicin	–	7 (7.3)	**–**
Extensively drug-resistant strains^a^	32 (66.7)	31 (32.3)	**<0.01**
Colistin MIC ≤ 2 μg/ml	28 (58.3)	64 (66.7)	0.42
Gentamicin MIC ≤ 2 μg/ml	15 (31.3)	72 (75)	**<0.01**
Tigecycline MIC ≤ 1 μg/ml^b^	16 (47.1)	58 (60.4)	0.27
Suboptimal MIC values^c^	10 (20.8)	14 (14.6)	0.5

Bold data are significant

*DC* double carbapenem, *ST* standard treatment, *CR* carbapenem resistant, *MIC* minimal inhibitory concentration

^a^Strains resistant to at least one agent in all but two or fewer usually active antimicrobial categories

^b^MIC values were available in 130 patients: 34 pts (DC group) and 96 patients (ST group)

^c^Gentamicin (4 μg/ml), tigecycline (2 μg/ml)

More than 90% of detected *Kp* produced class A carbapenemases (KPC), while the rest of the isolates harbored genes for class B metallo-β-lactamases and class D carbapenemases (OXA-48). About 40% of the isolated CR-*Kp* was resistant to either colistin or tigecycline (colistin sensitivity: 58.3% in the DC group vs 66.7% in controls, *p* = 0.42; tigecycline: 47.1% in the DC group vs 60.4% in controls, *p* = 0.27). Gentamicin sensitivity was significantly lower in patients receiving the ST regimen compared with the DC group (75% vs 31.3%, *p* < 0.01). Furthermore, 24 strains (16.7%) showed suboptimal MIC values for at least one rescue antimicrobial molecule (pharmacological and microbiological details presented in Table 2).

### Outcomes and predictors of mortality

Twenty-eight-day mortality was significantly higher in patients receiving ST compared with those who were treated with the DC regimen (47.9% vs 29.2%, *p* = 0.04). Despite a trend toward a better outcome in the DC group, the rates of clinical cure and microbiological eradication did not significantly differ between the groups. Overall, considering the 90-day mortality after infection, 50% of patients in the DC group were alive compared with 39.6% in controls, but despite such a reduction trend the difference was nonsignificant (Table 1).

**Fig. 1:**
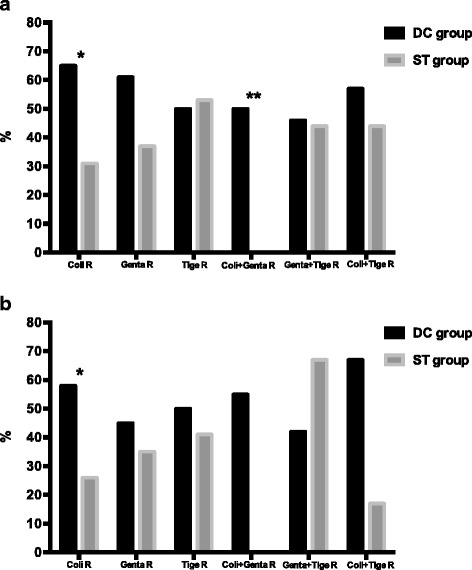
**a** Clinical cure rate according to antibiotic resistance. *p = 0.03, **p = 0.05. **b** Microbiological eradication rate according to antibiotic resistance. *p = 0.04. *R* resistance, *DC* double carbapenem, *ST* standard treatment, *Coli* colistin, *Genta* gentamicin, *Tige* tigecycline

However, stratifying the population according to the pattern of microbiological resistance, we found that clinical cure and microbiological eradication associated with DC were significantly higher in patients infected with CR-*Kp* resistant to colistin (clinical cure: 13/20 (65%) vs 10/32 (31.3%), *p* = 0.03; microbiological eradication: 11/19 (57.9%) vs 7/27 (25.9%), *p* = 0.04). Clinical cure was also higher for strains resistant to both gentamicin and colistin (clinical cure: 6/12 (50%) vs 0/6 (0), *p* = 0.05; microbiological eradication: 6/11 (54.5%) vs 0/5 (0), *p* = 0.09). No significant between-group differences were found in patients with germs resistant to either gentamicin or tigecycline, or to both gentamicin and tigecycline and to both colistin and tigecycline. Five infections were due to pan-resistant strains (three pneumonia and two complicated intra-abdominal infections): these were treated with the DC regimen, with a 40% rate of clinical cure and microbiological eradication (2/5) (Fig. 1).

**Fig. 2:**
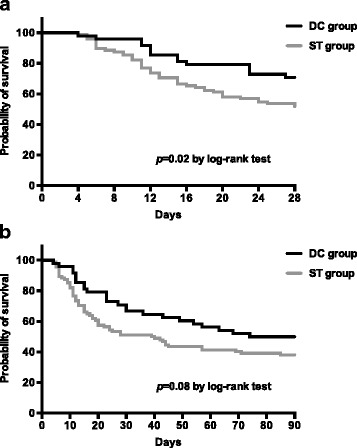
Kaplan–Meier curves showing the impact of DC therapy (*black line*) versus ST (*gray line*) on **a** 28-day mortality and **b** 90-day mortality. *DC* double carbapenem, *ST* standard treatment

On univariate analysis (Table 3), deceased numbers of patients had higher SAPS II and SOFA scores compared with survivors. Acute kidney injury requiring CRRT, polymicrobial infections, and inappropriate initial antimicrobial treatment occurred more frequently in patients who died. In multivariable logistic regression analysis, the DC regimen was the sole independent factor associated with 28-day survival, while higher SAPS II and SOFA score were significantly associated with mortality (Table 4). The multivariate Cox-regression model showed 28-day survival to be associated with the DC regimen (OR 0.43, 95% CI 0.23–0.79), and the difference in survival between treatment groups was also found on Kaplan–Meier survival curve analysis (*p* = 0.02). Unfortunately, such survival benefit was not maintained at 90-day mortality (*p* = 0.08) (Fig. 2).

**Table 3:** Univariate analysis of factors associated with 28-day mortality

Variable	Number (%) of patients		Univariate analysis	
	Alive (*n* = 84)	Deceased (*n* = 60)	*p* value	OR (95% CI)
Demographics and comorbidities				
Age (years)	57.7 ± 15	61.7 ± 10	**0.08**	**1.02 (0.99–1.05)**
Males	58 (69.1)	35 (58.3)	0.19	0.63 (0.31–1.25)
CHF	24 (28.6)	21 (35)	0.41	1.34 (0.66–2.74)
COPD	14 (16.7)	9 (15)	0.79	0.88 (0.35–2.2)
CRF	8 (9.5)	7 (11.7)	0.68	1.25 (0.43–3.67)
Diabetes	25 (29.8)	23 (38.3)	0.28	1.47 (0.73–2.95)
CLD	8 (9.5)	10 (16.7)	0.21	1.9 (0.7–5.14)
Neoplasm	7 (8.3)	7 (11.7)	0.51	1.48 (0.48–4.38)
Immunosuppressive status	27 (32.1)	18 (30)	0.78	0.9 (0.44–1.85)
ICU stay before infection (days), median (IQR)	8.5 (1–21)	8 (1–23)	0.69	1 (0.98–1.01)
Duration of MV before infection (days), median (IQR)	6 (1–13.5)	5.5 (0.5–15)	0.79	1.01 (0.98–1.03)
Duration of vasopressors before infection (days), median (IQR)	2 (0–7.5)	2 (0–7)	0.68	0.99 (0.97–1.02)
Presenting features, type of infection, and therapy				
Medical admission	45 (53.6)	39 (65)	0.17	1.61 (0.81–3.18)
Surgical admission	29 (34.5)	15 (25)	0.22	0.63 (0.3–1.32)
Trauma admission	10 (11.9)	6 (10)	0.72	0.82 (0.28–2.4)
SAPS II score, median (IQR)	39 (34–50)	55 (45.5–63)	**<0.01**	**1.07 (1.04–1.08)**
SOFA score on occurrence of infection, median (IQR)	8 (5–9)	9 (8–11)	**<0.01**	**1.36 (1.17–1.57)**
SS on occurrence of infection	42 (50)	37 (61.7)	0.17	1.6 (0.82–3.16)
ARDS on occurrence of infection	21 (25)	20 (33.3)	0.28	1.5 (0.72–3.11)
CRRT on occurrence of infection	22 (26.2)	24 (40)	**0.08**	**1.88 (0.92–3.82)**
PCT on occurrence of infection (ng/ml), median (interval)	3.4 (0.71–7.5)	4.3 (2.3–8.6)	0.43	1 (0.99–1.01)
Type of infection and therapy				
Pneumonia	41 (48.8)	33 (55)	0.46	1.28 (0.66–2.49)
IAI	14 (16.7)	5 (8.3)	0.15	0.45 (0.15–1.34)
SSTI	8 (9.5)	4 (6.7)	0.55	0.67 (0.19–2.37)
UTI	6 (7.1)	6 (10)	0.54	1.44 (0.44–4.72)
CVC BSI	11 (13.1)	7 (11.7)	0.8	0.88 (0.32–2.41)
Primary BSI	9 (10.7)	7 (11.7)	0.86	1.1 (0.39–3.14)
Secondary BSI	30 (35.7)	19 (31.7)	0.61	0.83 (0.41–1.69)
Polymicrobial infection	8 (9.5)	15 (25)	**0.01**	**3.17 (1.25–8.06)**
Double carbapenem therapy	34 (40.5)	14 (23.3)	**0.03**	**0.45 (0.21–0.94)**
IIAT	26 (30.9)	27 (45)	**0.08**	**1.82 (0.92–3.63)**
Combination targeted therapy	48 (57.1)	39 (65)	0.34	1.39 (0.7–2.76)
Duration of active treatment (days), median (IQR)	14 (8–18.5)	10 (8–14.5	0.13	0.96 (0.93–1.01)

Data presented as *N* (%), unless otherwise indicated. Bold data are significant

*OR* odds ratio, *IQR* interquartile range, *SAPS* Simplified Acute Physiology Score, *SOFA* Sequential Organ Failure Assessment, *ICU* intensive care unit, *MV* mechanical ventilation, *SS* septic shock, *ARDS* acute respiratory distress syndrome, *CRRT* continuous renal replacement therapy, *PCT* procalcitonin, *CHF* chronic heart failure, *COPD* chronic obstructive pulmonary disease, *CRF* chronic renal failure, *CLD* chronic liver disease, *IAI* intra-abdominal infection, *SSTI* skin and soft tissue infection, *UTI* urinary tract infection, *CVC* central vascular catheter, *BSI* bloodstream infection, *IIAT* initial inappropriate antimicrobial therapy

**Table 4:** Multivariable logistic regression analysis of factors associated with 28-day mortality

Variable	*p* value	OR (95% CI)
SAPS II score	<0.01	1.08 (1.04–1.23)
SOFA score	≤0.01	1.36 (1.33–1.63)
Double carbapenem treatment	0.02	0.33 (0.13–0.87)

We included all variables in the multivariable logistic regression if they reached *p* ≤ 0.1 on univariate analysis. A stepwise selection procedure was used to select variables for inclusion in the final model

ROC curve analysis was used to assess the goodness of the final logistic regression model (AUC ± SE = 0.85 ± 0.034 with 95% CI 0.78–0.91; chi-square statistics *p* < 0.001)

*AUC* area under the curve, *OR* odds ratio, *ROC* receiver operating characteristic, *SAPS* Simplified Acute Physiology Score, *SOFA* Sequential Organ Failure Assessment, *SE* standard error

## Discussion

In this population of critically ill patients affected with severe invasive infections due to carbapenem-resistant *Klebsiella pneumoniae*, the use of a DC regimen including ertapenem reduced 28-day mortality. Furthermore, clinical cure and microbiological eradication were more frequent in patients treated with the DC for colistin-resistant CR-*Kp* infections. Our findings support recent data suggesting the clinical efficacy of a DC regimen for the treatment of CR-*Kp* infections [21, 33].

*Kp*-producing carbapenemases are resistant to all beta-lactams, and to most aminoglycosides, fluoroquinolones, and sulphonamides [9]. The few remaining options are colistin, tigecycline, fosfomycin, and gentamicin, which usually harbor high MIC values, and share a suboptimal pharmacokinetic profile in terms of distribution at many infection sites [7, 13, 34]. Nonetheless, the use of gentamicin and colistin is frequently limited due to the risk of renal toxicity, and experience with the novel beta-lactam/beta-lactamase inhibitor combinations (e.g., ceftazidime–avibactam) in this setting is exiguous [16]. The rationale behind the usefulness of a DC regimen is intriguing: ertapenem, binding with high affinity to the active site of *Kp* carbapenemase, would be able to prevent the hydrolysis of the coadministered drug (i.e., meropenem, imipenem, or doripenem), which may preserve its bactericidal activity against the infecting isolate [35]. Indeed, carbapenemase consumption by ertapenem means that a high concentration of the associated drug can be active in the infection site, where a lower amount of carbapenemases will be available to degrade the administered antibiotic.

The preliminary observation of DC efficacy by Bulik and Nicolau in 2011 [18] was confirmed by clinical experiences documenting the efficacy of the DC regimen, including ertapenem plus meropenem or doripenem, in patients treated for CR-*Kp* infections [19–21, 33, 36, 37]. Recently a large series by Souli et al. [19] reported encouraging data on the use of a DC regimen as the exclusive therapy for extensively or pan-drug-resistant CR-*Kp*, further summarizing the actual clinical evidence in the field. In their cohort, a relevant clinical success was reached (77.8%), and also in the pan-resistant group the clinical and microbiological success was comparable (78.5%). In their review of the literature, a trend toward DC efficacy was highlighted, although there were some discrepancies among series.

In our study, we aimed at enhancing such evidence by comparing a larger cohort of DC-treated patients with a matched cohort of similar patients.

Given residual antimicrobial susceptibility, more than half of our patients receiving DC (35 [72.9%]) were treated with another active drug, mainly colistin. This approach is reasonable, and recent data suggest that the coadministration of colistin may increase the in-vivo and in-vitro activity of DC, and should be preferred in critically ill patients where an early clinical response is warranted [33, 38]. Similarly, in line with expert opinion, 52 of 96 controls were managed with a combined targeted therapy [39–41]. A carbapenem-sparing regimen has been proposed by Sbrana et al. [39], observing that the administration of tigecycline plus gentamicin or colistin was effective for the treatment of 24 CR-*Kp* infections in trauma patients, with a 14% mortality rate, further suggesting the usefulness of this approach when tigecycline is administered at a higher than standard dose (i.e., 50 mg every 12 hours after a 100 mg loading dose) [42]. On the other hand, recent data advocate the efficacy of combining high dosages of meropenem with colistin or tigecycline for the treatment of *Acinetobacter* infections [41]. These antibiotic associations have been seen to reduce mortality in patients with life-threatening infections due to extensively drug-resistant *Acinetobacter* and *Klebsiella*, but the benefits of meropenem can be expected only in the presence of MIC values close to susceptibility breakpoints. This kind of effect did not seem to have influenced the outcome of our cohort treated with DC because all of the strains were resistant to meropenem. In any event, we also have to address the idea of limiting the use of colistin in settings where KPC infections are frequent, given the alarming increase of colistin resistance in high-endemicity areas [43, 44].

Of note, we found a longer duration of antibiotic therapy in the DC group: such a result may be interpreted in light of the clinical profile of the DC group, in which patients, despite a higher percentage of septic shock, bacteremia rate, and PCT levels, showed higher survival together with a longer period of ICU support.

Moreover, the discrepancy in the effect of DC therapy between 28-day and 90-day mortality may be explained by the higher clinical impact of the septic episode on the short-term outcomes, along with the role of potential unmeasured confounders that may have influenced the 90-day mortality.

Our study has several limitations. First, although both centers adopted an electronic medical record, due to the observational nature we cannot exclude that unmeasured confounders may have influenced the strong association between DC use and improved survival. Second, synergistic assays for confirming DC in-vitro efficacy were not done routinely, although CR-*Kp* strains were studied according to genetic testing. Third, we did not check plasmatic carbapenem concentrations, so we can only assume that by optimizing dosages and administration modalities we achieved bactericidal meropenem levels at the infection site. Fourth, due to the use of the Vitek system, we cannot exclude that a small proportion of strains could display meropenem MIC values closer to the susceptibility breakpoint. Finally, we did not use either fosfomycin or ceftazidime–avibactam [45] because both molecules were not available at our centers during the observation period.

Nonetheless, this is the largest observational study in critically ill patients in whom the use of DC was evaluated, and the first investigation in which such a strategy is compared with other rescue treatments (i.e., colistin, tigecycline, gentamicin), after matching for severity of the disease.

## Conclusion

In the daily challenge of managing critically ill patients with CR-*Kp* infections, therapeutic options are limited. This observational analysis shows that the association of ertapenem plus meropenem provides a survival benefit, particularly when colistin cannot be used. Larger prospective studies are needed to confirm these findings, and to define the role of this unconventional strategy.

## Additional files

Additional file 1: **Figure S1** showing a flow chart of the study inclusion process. *CR-*Kp carbapenem-resistant *Klebsiella* pneumoniae, *DC* double carbapenem, *ST* standard treatment. (PDF 92.6 kb)

## Abbreviations

ARDS: Acute respiratory distress syndrome
BSI: Bloodstream infection
CR-*Kp*: Carbapenem-resistant *Klebsiella pneumoniae*
CRRT: Continuous renal replacement therapy
DC: Double carbapenem
EUCAST: European Committee on Antimicrobial Susceptibility Testing
HAP: Hospital-acquired pneumonia
IAI: Intra-abdominal infection
ICU: Intensive care unit
MALDITOF: Matrix-assisted laser desorption ionization-time-of-flight
MIC: Minimum inhibitory concentration
MS: Mass spectrometry
OR: Odds ratio
ROC: Receiver operating characteristic
SAPS: Simplified Acute Physiology Score
SOFA: Sequential Organ Failure Assessment
SS: Septic shock
SSTI: Skin and soft tissue infection
ST: Standard treatment
TDM: Therapeutic drug monitoring
UTI: Urinary tract infection
VAP: Ventilator-associated pneumonia
