# Supplementary figures and images for: Double carbapenem as a rescue strategy for the treatment of severe carbapenemase-producing Klebsiella pneumoniae infections: a two-center, matched case–control study
Source: Crit Care. 2017 Jul 5;21:173. doi: 10.1186/s13054-017-1769-z (PMC5498909; doi:10.1186/s13054-017-1769-z)

**eFigure 1**

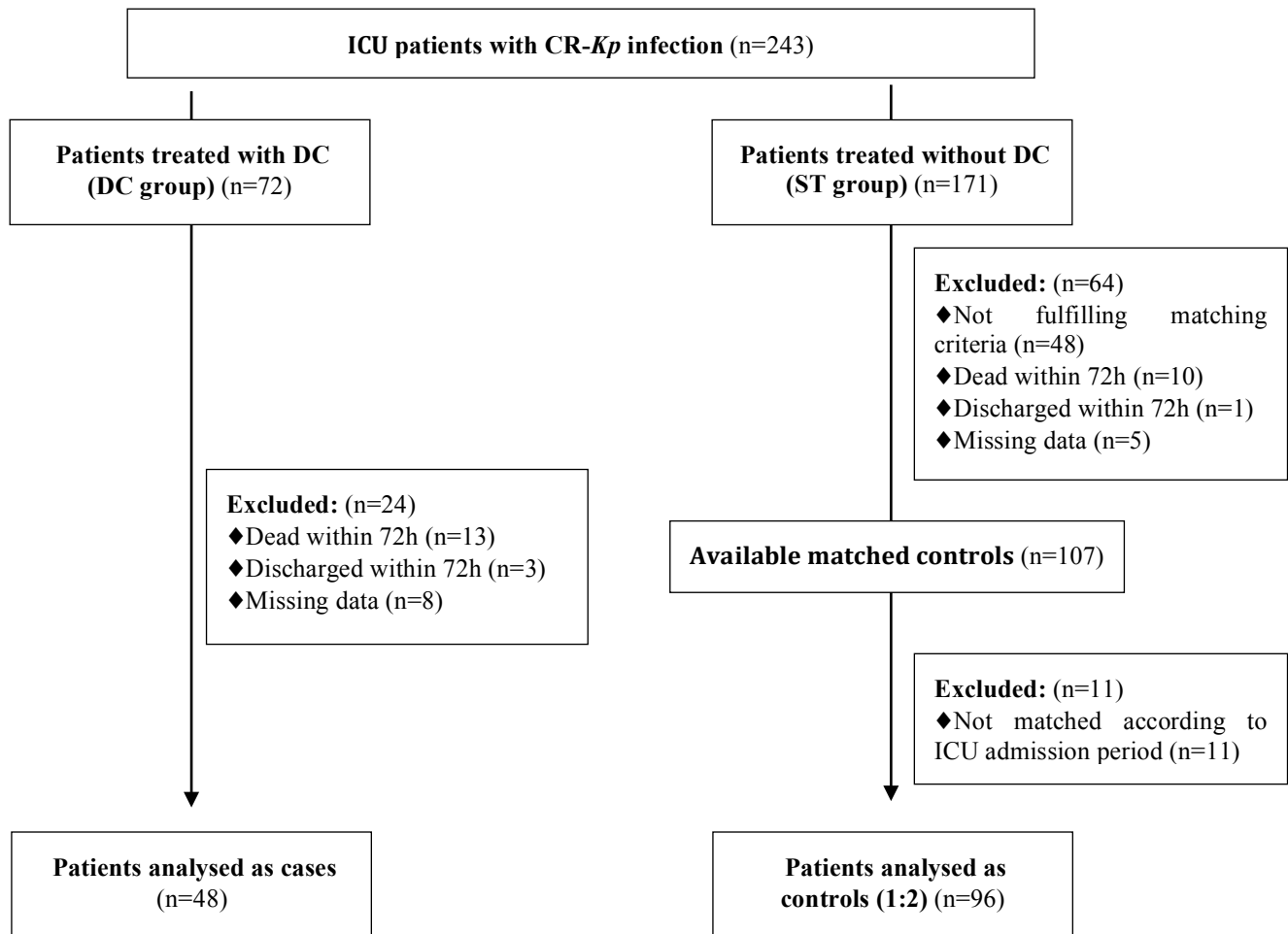

Supplement: Supplementary file 1 — Figure S1 showing a flow chart of the study inclusion process. CR-Kp carbapenem-resistant Klebsiella pneumoniae, DC double carbapenem, ST standard treatment. (PDF 92.6 kb) [file 13054_2017_1769_MOESM1_ESM.pdf]
